# Draft genome sequence of *“Burkholderia sola”* strains isolated in Mexico

**DOI:** 10.1128/mra.00805-24

**Published:** 2025-02-20

**Authors:** Leslie-Mariana Morales-Ruíz, Violeta Larios-Serrato, Paulina Estrada-de los Santos

**Affiliations:** 1 Departamento de Microbiología, Instituto Politécnico Nacional, Escuela Nacional de Ciencias Biológicas, Ciudad de México, Mexico; 2 Departamento de Bioquímica, Instituto Politécnico Nacional, Escuela Nacional de Ciencias Biológicas, Ciudad de México, Mexico; Department of Biological Sciences, Wellesley College, Wellesley, Massachusetts, USA

**Keywords:** *Burkholderia sola*, *Phaseolus vulgaris*, *Crotolaria pumila*, *Acacia* sp., *Coffea arabica*, Nodule-associated bacteria, rhizosphere-inhabiting microbes

## Abstract

This study presents the draft genomes of three strains of "*Burkholderia sola"* isolated from *Phaseolus vulgaris* L. trap plants and the rhizosphere of *Coffea arabica* L. in Mexico.

## ANNOUNCEMENT

**Fig 1:**
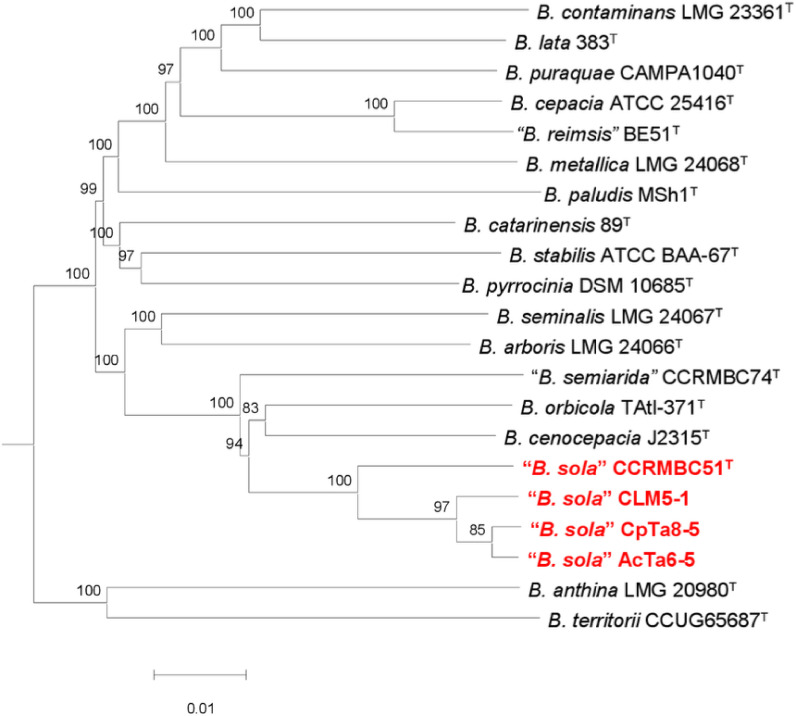
Phylogenomic tree of type strains of *Burkholderia* species and "*Burkholderia sola"* strains. Tree inferred with FastME 2.1.6.1 (14) from Genome BLAST Distance Phylogeny (GBDP) distances calculated from genome sequences. The branch lengths are scaled in terms of the GBDP distance formula d5. The numbers above branches are GBDP pseudo-bootstrap support values >60% from 100 replications, with an average branch support of 97.3%. The tree was rooted at the midpoint (15).

*Burkholderia*, belonging to the Class Betaproteobacteria, contains mainly human and animal pathogens, human opportunistic pathogens, and plant pathogens. Recently, a survey of bacteria from legume nodules and coffee rhizosphere revealed several genera, including *Burkholderia* (1). AcTa6-5 and CpTa8-5 strains were isolated from *Phaseolus vulgaris* trap plant root nodules inoculated with rhizospheric soil from *Crotolaria pumila* Ort. Chipil. and *Acacia* sp. Mill., respectively (1), and strain CLM5-1 was isolated from the coffee rhizosphere. The legume plants and soil were obtained from Tabasco (18°0′27.54″ N 93°19′5.664″ W) and Chiapas (14°58′59″ N 92°09′10″ W), Mexico. Five root nodules from *P. vulgaris* var. Negro Jamapa were surface disinfected with 10% commercial chloride for 10 min and washed five times with sterile water. The nodules were crushed with a plastic pestle, adding 40 mL of water. The suspension (15 µL) was inoculated onto yeast extract mannitol (YM) medium (5 g of mannitol) and incubated at 28°C for 3–5 days. For the coffee rhizosphere, 5 g was resuspended in sterile water, and then 50 µL was streaked in Ashdown medium (2) and incubated at 37°C for 72 h. The isolates were selected and stored in 30% glycerol at –70°C. The isolates were identified with universal primers 27F (5′-GTGCTGCAGAGACTTTGATCCTGGCTCAG-3′) and 1492R (5′-CACGGATCCTADGGGTACCTTACGACT-3′) (3) by amplifying the16S rRNA gene with a Taq DNA Polymerase (New England Biolabs). The gene was sequenced using the SANGER technology at Macrogen Inc. (https://www.macrogen.com/). To perform that, the isolates were grown in 5 mL of BSE medium, and the DNA was isolated with the CTAB protocol (4). The 16S rRNA gene sequences (MN830141, MN830137, and PP959370) were analyzed in the EzBioCloud Database (update 2023.08.23), which identified the strains as “*Burkholderia sola”* (GCA_029268985.1_m) (99.69 – 99.92% similarity). The species “*B. sola*” has been effectively published (5). However, the name has not been validly published in the International Journal of Systematic and Evolutionary Microbiology. Therefore, the name was written with quotation marks according to the List of Prokaryotic names with Standing in Nomenclature (6). The DNA for genome sequencing was obtained as described above. The DNA was sequenced by Novogene (https://www.novogene.com/us-en/) using the Illumina NovaSeq 6000 platform (150-bp paired-end reads; mean read length, 150 bp) with the NEBNext Ultra DNA library prep kit (New England Biolabs) and an insert size of 350 bp. For all the analyses, default parameters were used except where otherwise noted. Raw paired-end reads were trimmed with Trimmomatic v0.39 (7), to remove low-quality sequences (<15 phred) and Illumina adapters. Quality assessment was performed through FastQC v0.12.1 (8). *De novo* assembly was conducted using SPAdes v. 4.0 (9). Annotation was done using the standard operating procedure at the National Center for Biotechnology Information Prokaryotic Genome Annotation Pipeline v5.1 (10). Complete genome characteristics are shown in Table 1. Phylogenomic analysis placed “*B. sola*” strains close to *Burkholderia cenocepacia* J2315^T^ and *Burkholderia orbicola* TAtl-371^T^ (Fig. 1).

**TABLE 1:** Genome features of "*Burkholderia sola"* strains^*a*^^,^^*b*^

Feature	AcTa6-5	CpTa8-5	CLM5-1
Raw reads	8,410,110	8,949,520	9,726,548
Genome size (Mbp)	7,560,992	7,644,067	7,824,979
Number of contigs	102	104	971
N50 contig length (kb)	221,487	260,692	128,806
Genome coverage (X)	75	75	75
Completeness (%)	99.36	99.57	99.36
Contamination (%)	0.69	0.69	3.54
GC content (%)	67	67	67
Total genes	6,907	6,931	7,756
Coding genes	6,761	6,866	7,556
RNA genes	66	65	59
tRNA	57	56	52
rRNA (complete 5S, 16S, 23S)	1, 1, 1	1, 1, 1	1, 1, 1
Digital DNA: DNA hybridization (dDDH) to "*B. sola*" CCRMBC51 (%)	71.2	70.9	70.5
Average nucleotide identity (ANI) to "*B. sola*" CCRMBC51^T^ (%)	96.62	96.63	96.58
Genome accession	JBEWCI000000000	JBEWCH000000000	JBEWCG000000000
Genome assembly	ASM4053007v1	ASM4052998v1	ASM4053002v1
SRA	SRR29619567	SRR29619566	SRR29619565

^*a*^ dDDH was performed using the Genome-to-Genome Distance Calculator version 3.0, with formula 2 (11). The bacterial species demarcation criterion is 70% (12).

^*b*^ ANI was performed using the OrthoANIu web tool (13). The bacterial species demarcation criterion is 95%–96% (12).

## Data Availability

GenBank assembly and SRA numbers of “*B. sola*” strains are GCA_040530075.1, GCA_040529985.1, GCA_040530025.1, SRR29619567, SRR29619566, and SRR29619565. More accession numbers are included in Table 1.
